# Dysfunctional mitochondria elicit bioenergetic decline in the aged heart

**DOI:** 10.20517/jca.2023.50

**Published:** 2024-02-01

**Authors:** Pasquale Mone, Esther Densu Agyapong, Giampaolo Morciano, Stanislovas S. Jankauskas, Antonio De Luca, Fahimeh Varzideh, Paolo Pinton, Gaetano Santulli

**Affiliations:** 1 Department of Medicine (Division of Cardiology), Wilf Family Cardiovascular Research Institute, Einstein Institute for Aging Research, Albert Einstein College of Medicine, New York, NY 10461, USA.; 2 Department of Medicine and Health Sciences, University of Molise, Campobasso 86100, Italy.; 3 Department of Medical Sciences, University of Ferrara, Ferrara 44121, Italy.; 4 Maria Cecilia Hospital, GVM Care & Research, Cotignola 48033, Italy.; 5 Department of Mental and Physical Health and Preventive Medicine, Vanvitelli University, Naples 80100, Italy.; 6 Department of Advanced Biomedical Sciences, “Federico II” University, International Translational Research and Medical Education (ITME) Consortium, Academic Research Unit, Naples 80131, Italy.; 7 Department of Molecular Pharmacology, Einstein-Mount Sinai Diabetes Research Center (ES-DRC), Einstein Institute for Neuroimmunology and Inflammation (INI), Fleischer Institute for Diabetes and Metabolism (FIDAM), Albert Einstein College of Medicine, New York, NY 10461, USA.

**Keywords:** Autophagy, cardiac aging, fibrosis, mitochondria, mitophagy, neurohormonal systems, oxidative stress, RAAS, ROS, sympathetic nervous system

## Abstract

Aging represents a complex biological progression affecting the entire body, marked by a gradual decline in tissue function, rendering organs more susceptible to stress and diseases. The human heart holds significant importance in this context, as its aging process poses life-threatening risks. It entails macroscopic morphological shifts and biochemical changes that collectively contribute to diminished cardiac function. Among the numerous pivotal factors in aging, mitochondria play a critical role, intersecting with various molecular pathways and housing several aging-related agents. In this comprehensive review, we provide an updated overview of the functional role of mitochondria in cardiac aging.

## INTRODUCTION

Aging is known to induce alterations in the heart, irrespective of specific cardiovascular risk factors like diabetes or hypertension, resulting in cardiac remodeling and functional changes.

## CLINICAL OBSERVATIONS

Standard echocardiography reveals modifications in cardiac dimensions and wall thickness. The prevalence of left ventricular hypertrophy (LVH) increases with age, even in the absence of hypertension^[1–5]^. Diastolic dysfunction is a prevailing feature, evidenced by compromised LV filling in early diastolic phase (peak E wave), potentially due to fibrosis and reduced ventricular elasticity^[6]^. Atrial contraction (A wave) increases with age, contributing to atrial hypertrophy and heightened atrial fibrillation risk^[7–10]^. The E/A ratio, reflecting diastolic LV filling dynamics, declines significantly with age, indicating an increased reliance on late diastolic filling, suggestive of diastolic dysfunction^[9,11]^. Torsion, a characteristic rotational deformation during systole, increases with age, potentially indicating subendocardial dysfunction due to fibrosis^[12,13]^.

## NEUROHORMONAL SYSTEMS AND CARDIAC AGING

The adrenergic system and the renin-angiotensin-aldosterone system (RAAS) play decisive roles in cardiac remodeling during aging. Cardiac aging involves progressive collagen deposition, leading to interstitial fibrosis and altered cardiac elasticity, predominantly affecting diastole over systole. Elevated intracardiac concentrations of Angiotensin II (Ang II) accompany aging, substantially contributing to functional, molecular, and structural modifications consistent with Ang II effects^[14,15]^. In fact, inhibiting Ang II signaling by enalapril or losartan has been shown to extend lifespan and delay age-related cardiovascular pathologies^[16–19]^.

Cardiac aging also features a substantial depletion of autonomic fibers and reduced cardiac and circulating levels of brain-derived neurotrophic factor (BDNF)^[20]^. The latter rules basal myocardial contractility/relaxation^[21]^ and metabolism^[22]^.

The adrenergic system, particularly through β adrenergic receptors (βARs), is intimately linked to cardiac aging, a process marked by a decline in βAR sensitivity and density, which is fairly consistent across species^[9,23,24]^.

Overall, aging exhibits an uncontrolled activation of the adrenergic system, impacting βARs, their subtypes (β1, β2, and β3), and their localization in the heart. Spatially restricted cAMP production due to the unique localization of β2AR in cardiac cells may be disrupted in aging, affecting calcium-dependent proteins and myofilament contraction^[25–28]^. Such a remodeling might contribute to the typical features of the aging heart. Additionally, the disruption of adenylyl cyclase type 5 (AC5), a βAR downstream signaling enzyme, has been shown to protect against age-dependent cardiac issues^[29–31]^.

The adrenergic system is implicated in metabolic alterations, especially in heart failure^[32,33]^. Cardiac bioenergetic changes with age, as evidenced, for instance, by alterations in the creatine kinase enzyme system and by increased catecholamines, eventually affecting mitochondrial function^[34–36]^. Indeed, chronic βAR stimulation is known to induce mitochondrial membrane depolarization and apoptosis, further emphasizing the convoluted connections between adrenergic signaling and mitochondrial function during cardiac aging^[37–40]^.

## MITOCHONDRIAL DYSFUNCTION IN CARDIAC AGING

Mitochondria, often referred to as cell powerhouses, generate adenosine triphosphate (ATP) through oxidative phosphorylation, a meticulously regulated process for maintaining cellular energy balance^[41,42]^. The mitochondrial electron transport chain (ETC) contributes 80%−90% of ATP in most mammalian tissues, making mitochondrial dysfunction detrimental due to reduced ATP production, indispensable for biological functions^[43,44]^. Aging leads to alterations in ETC components, contributing to a number of age-related conditions^[45–48]^. Hence, one of the consequences of the aging process is the drop in the efficiency of this energy production mechanism, leading to a decline in ATP production and subsequent cellular energy deficits^[49,50]^. In fact, dysfunctional mitochondria are increasingly associated with aged cardiovascular tissues.

Proper substrate utilization is imperative for the myocardium to fulfill its function, primarily relying on ATP (re-)synthesis through fatty acid oxidation within mitochondria^[51]^. The myocyte employs additional pathways, such as glycolysis, creatine kinase, and adenylate kinase, in response to high ATP demand. During increased work, glycogen, glucose, and phosphocreatine are utilized to meet ATP demand, maintaining constant ATP levels. The efficiency of ATP production varies depending on substrate oxidation, with fatty acid oxidation producing more ATP on a molar basis^[52]^. This metabolic plasticity diminishes in chronic pathologies like congestive heart failure, impacting myocardial oxygen efficiency and causing intracellular ATP depletion.

Spare respiratory capacity, the ability to increase ATP production during heightened demand or reduced fuel supply, is crucial for cellular function and survival. Mitochondrial plasticity involves the efficiency of mitochondrial coupling and provides increased respiratory capacity under stress conditions. This phenomenon is particularly significant in ischemic injury and situations of augmented energy demand such as sepsis, endurance exercise, trauma, or heart failure^[53]^. Mitochondrial plasticity is decreased in patients with insulin resistance and in elderly individuals, impacting cell function^[54]^. In the heart, aging leads to a decline in mitochondrial oxidative phosphorylation function, especially in state 3 respiration, related to electron transport complexes I and IV^[55]^. Increased electron leakage and mitochondrial ROS production contribute to oxidative damage, particularly affecting intrafibrillar mitochondria. Deficient mitochondrial energetics, altered Ca^2+^ homeostasis, and excessive ROS generation contribute to reduced stress adaptability and augmented vulnerability to disease in the aged myocardium^[56,57]^. Interconnected communication between the sarcoplasmic reticulum and mitochondria supports local signal transduction and ionic exchange^[58–62]^. Mitochondrial Ca^2+^ uptake plays fundamental roles in energy supply-demand matching and antioxidative defenses. Aging negatively impacts mitochondria-SR communication, potentially involving an impaired Ca^2+^ transmission and decreased physical interaction between ryanodine receptors (RyR) and mitochondrial VDAC (voltage-dependent anion channel)^[63,64]^.

Insulin receptor signaling is central in mediating processes boosting glucose uptake in cardiomyocytes, and cardiac insulin resistance is known to contribute to ventricular dysfunction by favoring fatty acid utilization^[65]^. Insulin resistance is associated with metabolic inefficiency and impaired mitochondrial fitness^[66,67]^, leading to contractile dysfunction. Mitochondrial abnormalities, reduced expression of oxidative phosphorylation regulators, increased reactive oxygen species (ROS), and compromised mitochondrial biogenesis and myocyte energetics are observed in senescent hearts. Molecular modifications characteristic of heart failure, such as shifts in membrane receptor signaling, survival-kinase signal transduction, and mitochondrial dysfunction/cell death, are also observed in the senescent heart^[68]^.

## MITOCHONDRIAL OXIDATIVE STRESS IN AGING

In the aging heart, mitochondria become a significant source of ROS. Their excessive production leads to a consequent imbalance with the vasoprotective bioavailability of nitric oxide^[69]^. ROS, also known as oxygen-containing reactive chemical species, represent a variety of oxidizing chemical compounds ranging from the highly reactive hydroxyl group to hydrogen peroxide^[70]^.

ROS production leads to lipid peroxidation and the onset of DNA damage, which contribute to accelerating the processes linked to cardiac aging. Within intact cardiomyocytes, major sources of ROS commonly derive from the mitochondrial OXPHOS complexes I (i.e., NADH dehydrogenase) and III (i.e., cytochrome bc1 complex), which are part of the electron transport chain (ETC)^[71]^.

In the ETC, NADH and FADH_2_ supply electrons to complex I and complex II (i.e., succinate dehydrogenase). These electrons are subsequently transmitted to complex III via ubiquinone (i.e., CoQ, coenzyme Q) and then progress through complex IV (i.e., cytochrome c-oxidase) until reaching molecular O_2_, which represents the final acceptor^[72]^. The sources of origin of ROS are multiple, but their main production is attributed to reverse electron transport via complex I through means of high proton driving force or low CoQ availability^[73]^. Furthermore, ROS can emerge from single electrons escaping from the ETC in an inactive process called electron scattering^[74]^. The escape of these electrons leads them to interact with O_2_, reducing it to generate a very unstable superoxide anion (O_2_‒ or O_2_·‒)^[75]^. These cytotoxic oxygen intermediates are metabolized by glutathione peroxidase (GPX), catalase (Cat), and superoxide dismutase (SOD)^[76–78]^, antioxidant systems that are extremely efficient in healthy cardiomyocytes. To counteract this ROS production, mitochondria employ this network of ROS-scavenging enzyme systems, which attempt to mitigate the excessive stress produced^[79–81]^. SOD catalyzes the conversion of O_2_‒ into hydrogen peroxide (H_2_O_2_) that is then decomposed into oxygen and water by Cat; GPX can convert hydroxyl radicals and peroxides into non-toxic forms^[82]^. However, H_2_O_2_ easily interacts with metal atoms, giving rise to the extremely reactive hydroxyl radical (OH•)^[83]^. The ubiquitous OH• strips electrons from nucleic acids, lipids, and proteins, causing damage to the cardiomyocytes. Studies in aged rat hearts found that steady-state levels of O·−2 and H_2_O production were increased compared to their adult counterparts^[84]^

Mitochondria engaged in respiration continually generate H_2_O_2_. If the production surpasses scavenging capacity, H_2_O_2_ emission takes place, posing a threat to cellular functions^[71,85–88]^. Peroxiredoxin-3, a mitochondrial peroxidase, has been shown to mitigate H_2_O_2_ by converting it to H_2_O, utilizing reducing equivalents from NADPH provided by thioredoxin-2 and thioredoxin reductase-2^[89]^.

The physiologic pattern of ROS production becomes exacerbated in aging because of detrimental attacks within the cardiomyocyte over time^[90–93]^. It also determines the accretion of dysfunctional organelles and damaged proteins, which contribute to increasing the incidence of many age-related cardiovascular diseases^[94,95]^.

Over the years, several studies have been conducted on a murine model overexpressing human antioxidant catalase (mCAT)^[96,97]^. This overexpression increased lifespan due to decreased oxidative stress and led to phenotypes with reduced cardiac aging, substantiated by improved contractile function and mitigated hypertrophy^[98]^. The mCAT mouse models also displayed attenuated mitochondrial H_2_O_2_ toxicity, decreased oxidative DNA damage, and decreased accumulation of mutations in mitochondrial DNA (mtDNA)^[99]^.

Cardiomyocytes are long-lived postmitotic cells that can live for many years: they have a limited capacity for regeneration and a rare propensity for malignant transformation^[100,101]^. Intriguing theories related to mitochondrial aging in the heart are related to the high mutation rate and limited repair capacity of mtDNA, compromising the integrity of the mitochondrial genome^[102,103]^. As a consequence, in many cardiac cells undergoing the aging process, mitochondrial functions are impaired and their ability to generate energy is progressively lost, counterbalanced by increased generation of ROS^[104]^.

The presence of deletions and point mutations in mtDNA has been reported to increase with age in various tissues including the myocardium^[105]^. A previous study has demonstrated increased levels of H_2_O_2_ in subsarcolemmal mitochondria (SSM) and increased levels of oxidative stress in interfibrillar mitochondria (IFM) in isolated hearts of aged rats compared to young ones^[106]^.

## MITOCHONDRIA AND INFLAMMAGING

By activating different redox signaling pathways, mitochondrial ROS are known to contribute to chronic vascular inflammation in aging^[107]^. Chronic inflammatory processes are recognized as contributing factors to age-related cardiovascular disorders such as vascular cognitive impairment, heart failure, peripheral artery disease, and stroke^[108]^. For instance, when inflammation occurs, there are many pro-inflammatory changes in the endothelial phenotype. They include an increase in cell adhesion molecules, augmented endothelium-leukocyte interactions, and changes in the secretion of pro-inflammatory molecules. Previous investigations have shown that advanced age induces modifications in cytokine profiles and triggers the expression of pro-inflammatory genes in cardiac muscle, in the perivascular adipose tissue, and in the walls of large arteries^[109,110]^. The activation of t NF-κB plays an essential role in pro-inflammatory modifications and endothelial activation in the vasculature associated with aging both in humans^[111]^ and in laboratory mouse models^[107]^.

Emerging evidence indicates that mitochondria-associated factors may activate a number of innate immune mechanisms that modulate host defenses, such as interferon (IFN)-dependent antiviral responses and inflammasome-dependent pro-inflammatory cytokines^[112]^.

## AUTOPHAGY AND AGING

Autophagy, a fundamental process in organelle and protein degradation, plays a key role in programmed cell death^[113–116]^. Three main types of autophagy have been described: macroautophagy, microautophagy, and chaperone-mediated autophagy (CMA)^[117–119]^. Macroautophagy involves the sequestration of portions of cytoplasm within autophagosomes, double-membrane-enclosed vacuoles, which then fuse with lysosomes for subsequent degradation^[120]^. Microautophagy, on the other hand, does not require sequestration; small portions of cytoplasm enter lysosomes via invaginations within the limiting membrane^[121]^.

CMA does not involve vesicular traffic. In CMA, cytosolic proteins with specific peptide sequence motifs (KFERQ) are recognized by molecular chaperones. This substrate-chaperone complex then binds to Lamp2a (lysosome-associated membrane protein type 2a), a receptor on the lysosomal membrane, facilitating the entry of proteins into the lysosomal lumen^[121,122]^.

## CARDIOPROTECTIVE EFFECTS OF AUTOPHAGY

Numerous studies have demonstrated that inducing autophagy in the heart has cardioprotective effects^[123–129]^. Additionally, autophagy tends to decrease during aging, and interventions that enhance autophagy have shown promise in reducing age-related cardiac disorders and extending lifespan^[130–132]^.

However, excessive autophagy might prove harmful, as it may lead to a disproportionate degradation of essential cellular components, ultimately resulting in cell death^[133,134]^.

## MAIN INTRACELLULAR MECHANISMS UNDERLYING AUTOPHAGY

Specifically, various intracellular mechanisms play a critical role in either inhibiting or promoting autophagy, a process intimately associated with aging^[114,135]^. For instance, mTOR acts as a sensor^[136]^ for energy metabolism and nutrient availability, negatively regulating autophagy: mTOR forms two complexes, mTORC1 and mTORC2; mTORC1, linked to aging, inhibits autophagy via phosphorylation of the ULK1-Atg13-FIP200 complex^[137–139]^. Growth factors (e.g., insulin-like growth factor, IGF) are known to activate mTOR^[140]^, suppressing autophagy. In contrast, mTOR inhibition, achieved by drugs like rapamycin, mimics starvation conditions and promotes autophagy^[141–143]^. AMPK (AMP-Activated Protein Kinase) is a well-established energy sensor that activates autophagy by inhibiting mTORC1 through Raptor phosphorylation and TSC2 phosphorylation; it also directly binds to the ULK1 complex, promoting autophagy^[144,145]^. The cross-talk between ULK1, AMPK, and mTORC1 finely modulates synthetic and catabolic processes^[145,146]^. FOXO Proteins (Forkhead Box O) positively regulate autophagy by activating the transcription of specific genes; they are inhibited by nuclear AKT in the presence of growth factors, while they are activated by AMPK during low energy levels^[114,147]^.

SIRT1, a member of the Sirtuin family, regulates intracellular bioenergetics. It deacetylates FoxO1, promoting autophagy, and also deacetylates ATG proteins. SIRT1 is cardioprotective, but chronic elevated levels can be detrimental, leading to oxidative stress, apoptosis, and cardiomyopathy^[148–152]^. Proper regulation of SIRT1 is necessary for maintaining cardiac function^[150,153,154]^. The Sirtuin family is also essential for the regulation of the mitochondrial unfolded protein response (mtUPR), one of the first stress-protective responses started by mitochondrial dysfunction^[155]^, which clears or repairs misfolded proteins in order to mitigate the damage^[156–158]^.

Equally important, the induction of endoplasmic reticulum stress (ER stress) has been shown to damage the ETC in aged cardiac mitochondria, in a process regulated by mitochondria-localized calpain 1 and calpain 2^[159]^.

Eventually, oxidative damage towards the cardiovascular system leads to functional failure of the mitochondrion^[160–164]^. Hence arises the necessity for the cell to eliminate these dysfunctional organelles in order to preserve cellular homeostasis^[165,166]^.

## RECEPTOR-MEDIATED MITOPHAGY

Damaged mitochondria are selected for degradation via a specific form of autophagy (mitophagy)^[141,167]^. Receptor-mediated mitophagy relies on receptors located on the outer mitochondrial membrane (OMM). These receptors, including Bcl2/adenovirus E1B 19-kDa interacting protein 3 (BNIP3), Bcl-2-like protein 13 (Bcl2L13/Bcl-Rambo), NIX/BNIP3L, FUN14 domain-containing 1 (FUNDC1), and FK506-binding protein (FKBP8), are anchored to the OMM and directly bind to LC3, guiding the targeted organelle to the autophagosome without ubiquitination^[168–170]^. Limited information exists on the exact role of mitophagy players during aging, but it is known that BNIP3 is upregulated in aged hearts^[171]^. Notably, NIX and BNIP3 may transition to maladaptive inducers of cell death in age-induced cardiac stress, deviating from their role in promoting mitochondrial quality control^[172,173]^.

## PARKIN AND CARDIAC AGING

The PINK1-Parkin mitophagy pathway (PTEN-induced putative kinase protein 1, Parkinson Protein 2) involves targeted degradation of damaged mitochondria^[174]^. PINK1, under normal conditions, translocates and is degraded within the mitochondria. PINK1 and Parkin target and degrade damaged and poorly functioning mitochondria^[175,176]^: following oxidative stress-induced membrane depolarization, PINK1 accumulates, recruiting Parkin, which ubiquitinates damaged mitochondria^[177,178]^. Autophagosomes enclose these mitochondria, fusing with lysosomes to initiate degradation. Loss of Parkin function hinders effective clearance, potentially triggering further mitochondrial fission^[175]^. Many investigations focused on vascular aging have confirmed the role of mitophagy mediated by PINK1 and Parkin. A reduction in Parkin expression was detected in the aorta of aged mice and was then correlated with increased superoxide production and aortic stiffness^[179]^. Theablation of Parkin in aged mice was shown to cause accumulation of abnormal mitochondria in the heart, and interestingly, mice lacking Parkin showed enhanced aging compared to controls in which the protein was normally expressed^[180]^. On the contrary, overexpression of Parkin in cardiomyocytes ameliorated mitochondrial health, slowing down cardiac aging^[181]^.

Over the years, clinical investigations have confirmed the preclinical hypotheses demonstrating reduced cardiac expression of PINK1 in elderly patients with heart failure compared with healthy individuals^[182]^.

## AGING AND MITOCHONDRIAL DYNAMICS

Mitochondrial dynamics, including fusion and fission events, are integral to maintaining mitochondrial function. Imbalances in these dynamic processes have also been implicated in cardiovascular aging^[100,168,183,184]^. Excessive fission results in fragmented mitochondria, promoting cellular dysfunction, while impaired fusion compromises mitochondrial networking and bioenergetic efficiency^[183,185]^.

Specific dynamin-related GTPases play functional roles in the mitochondrial fusion process: optic atrophy 1 (OPA1) for inner membrane fusion and mitofusins (MFN1 and MFN2) for outer mitochondrial membrane (OMM) fusion^[186–188]^.

Fission leads to the division of mitochondria into two separate organelles. Drp1 (dynamin-related protein 1) is the master regulator of mitochondrial fission. Upon activation, Drp1 translocates from the cytosol to the mitochondria^[189–191]^ and then it forms a coiled ring with an internal diameter of ~20 nm^[192]^. All the morphological alterations caused by inadequate fission and fusion processes play a role in cardiac aging, resulting in fibrosis, diastolic dysfunction, and ventricular hypertrophy. A reduced expression of MFN1-MFN2 has been reported in 25-month-old rats, with an increase in Opa1 and Drp1 in 36-month-old rats^[193]^. A shift towards mitochondrial fusion exacerbates cellular senescence, as highlighted by studies of senescence promoting Fis1 knockdown^[194]^.

Furthermore, alterations in mitochondrial fusion accelerate cardiac aging. Opa1^+/−^ mice showed fragmented mitochondria and an impaired myocardial function^[195]^. Similarly, Mfn2-deficient mice exhibited impaired left ventricular function by 17 months^[196]^. A balance between the mechanisms of mitochondrial fusion and fission would allow the cardiac tissue to be preserved from the onset of age-related anomalies^[197–200]^.

## CONCLUSIONS

**Figure 1.**
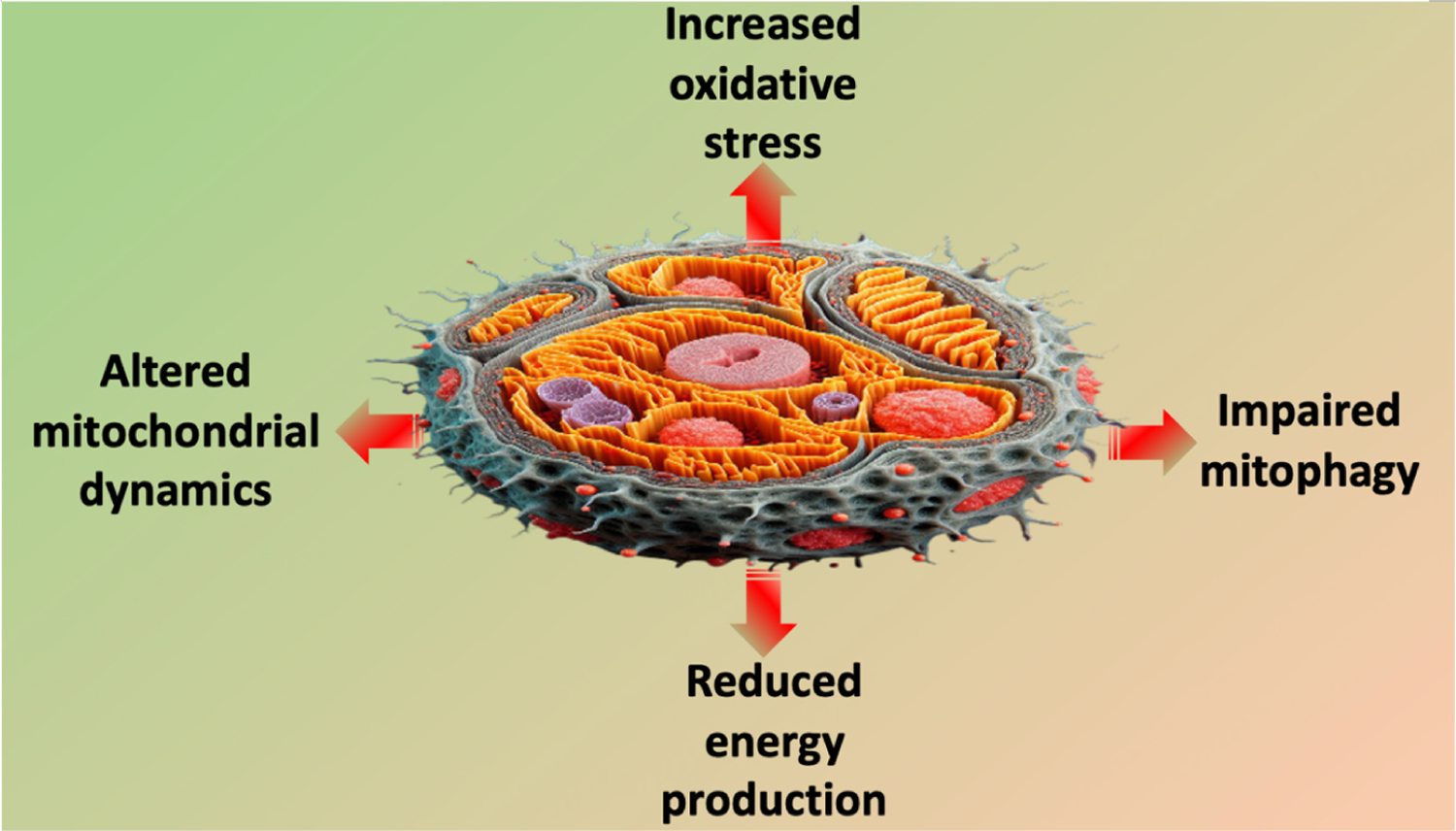
Main mitochondrial alterations in cardiovascular aging.

Unraveling the molecular intricacies of the relationship linking mitochondria to myocardial health provides a foundation for developing targeted interventions to mitigate age-related cardiovascular decline [Figure 1]. As research in this field continues to evolve, the prospect of therapeutic strategies aimed at preserving mitochondrial fitness and promoting cardiovascular health in aging populations becomes increasingly promising. Therefore, strategies aimed at improving mitochondrial function in older patients may be cardio-protective.
